# Exposure to a youthful circulaton rejuvenates bone repair through modulation of β-catenin

**DOI:** 10.1038/ncomms8131

**Published:** 2015-05-19

**Authors:** Gurpreet S. Baht, David Silkstone, Linda Vi, Puviindran Nadesan, Yasha Amani, Heather Whetstone, Qingxia Wei, Benjamin A. Alman

**Affiliations:** 1 Department of Developmental and Stem Cell Biology, Hospital for Sick Children, University of Toronto, Toronto, Ontario M5G 0A4, Canada; 2 Department of Orthopedics, Duke University, Durham, North Carolina 27710, USA

## Abstract

The capacity for tissues to repair and regenerate diminishes with age. We sought to determine the age-dependent contribution of native mesenchymal cells and circulating factors on *in vivo* bone repair. Here we show that exposure to youthful circulation by heterochronic parabiosis reverses the aged fracture repair phenotype and the diminished osteoblastic differentiation capacity of old animals. This rejuvenation effect is recapitulated by engraftment of young haematopoietic cells into old animals. During rejuvenation, β-catenin signalling, a pathway important in osteoblast differentiation, is modulated in the early repair process and required for rejuvenation of the aged phenotype. Temporal reduction of β-catenin signalling during early fracture repair improves bone healing in old mice. Our data indicate that young haematopoietic cells have the capacity to rejuvenate bone repair and this is mediated at least in part through β-catenin, raising the possibility that agents that modulate β-catenin can improve the pace or quality of fracture repair in the ageing population.

## Abstract (web summary)

The reparative capacity of tissues decreases with age. Here, Baht *et al*. perform parabiosis experiments and provide evidence for the existence of a soluble factor produced by young, but not old, haematopoietic cells that is capable of improving fracture repair in old mice.

## Graphical abstract

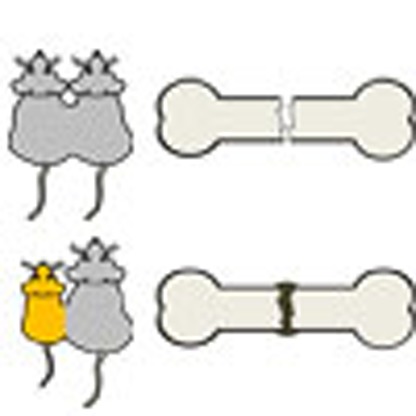

The capacity for tissue repair and regeneration diminishes with age. Accordingly, bone repair and related processes, such as osseous integration of implants, occurs at a slower pace in older patients than in younger patients. This results in the need for additional surgical procedures to attain proper bone healing and is associated with increased rates of morbidity and mortality in the elderly^1^,^2^. Likewise, *in vitro* differentiation of bone marrow stromal cells (BMSCs) to osteoblasts is less effective in BMSC cultures from older patients than from younger patients^3^,^4^. Similar to humans, fracture repair and osteoblast differentiation are less efficient in older mice than in younger mice^5^. The cause of this difference in bone repair and osteogenic potential with age is not known. Work investigating age-dependent deficiencies in muscle regeneration identified that a youthful circulation can rejuvenate aged-muscle repair^6^,^7^,^8^. β-catenin signalling was implicated in this muscle rejuvenation^9^,^10^,^11^,^12^. Interestingly, precise regulation of β-catenin is critical for successful bone fracture repair^13^,^14^,^15^,^16^,^17^ as well as differentiation of mesenchymal progenitors to osteochondral lineages^14^,^18^,^19^. β-catenin levels during fracture repair are transiently modulated. During early stages of fracture repair (first 7 days) mesenchymal progenitor cells are committing to an osteochondral progenitor lineage. For proper commitment of mesenchymal cells to an osteochondral progenitor, tight control of β-catenin levels is required. On commitment to an osteochondral progenitor, increased levels of β-catenin acts as a positive pressure for osteoblastic differentiation. Indeed, previous lines of work from our laboratory and others show that increased levels of β-catenin lead to higher bone density and enhanced osteoblastic differentiation; however,this applies to a differentiated, osteoblastic cell population^14^,^17^,^20^. Increased levels of β-catenin during the early phases of repair, or before a mesenchymal progenitor becoming an osteochondral progenitor, leads to differentiation to fibroblastic phenotype, inhibiting fracture repair^14^. Thus, β-catenin levels need to be precisely regulated for effective differentiation to osteoblasts during fracture healing, with higher or lower levels impairing normal healing^13^,^14^,^15^,^16^,^17^,^18^,^19^.

We sought to determine the effect of mesenchymal cell age and the age of the circulation on bone regeneration and osteoblast differentiation to understand the mechanism by which repair rate is slowed. Exposure to a young circulation rejuvenated *in vivo* bone-fracture repair and *in vitro* osteoblast differentiation. This rejuvenation was independent of young osteoblastic cells and relied on a signal to the endogenous, aged osteoblasts to increase bone matrix deposition and mineralization. A similar rejuvenation was observed upon engraftment of young bone marrow (young haematopoietic cells) into aged mice. In cell culture conditions, media conditioned by young BMSCs were able to rejuvenate osteoblastogenesis of old cells. Modulation of the β-catenin protein level was found to be required for this rejuvenation. Decreasing levels of β-catenin early during fracture repair was able to rescue the aged fracture-repair phenotype.

This work demonstrates that diminished tissue-regeneration capacity is at least partly dependent on the regulation of β-catenin levels in the cell. With age, signalling of the canonical β-catenin pathway increases^11^,^12^. During tissue regeneration, this heightened level of β-catenin signalling leads to a fibrotic response during bone-tissue repair. We rescued the aged-bone regeneration phenotype through modulation of basal β-catenin levels during early stages of injury. This raises the potential use of pharmacological agents that modulate β-catenin to enhance fracture repair in ageing.

## Results

### Youthful circulation rejuvenates aged bone healing

**Figure 1:**
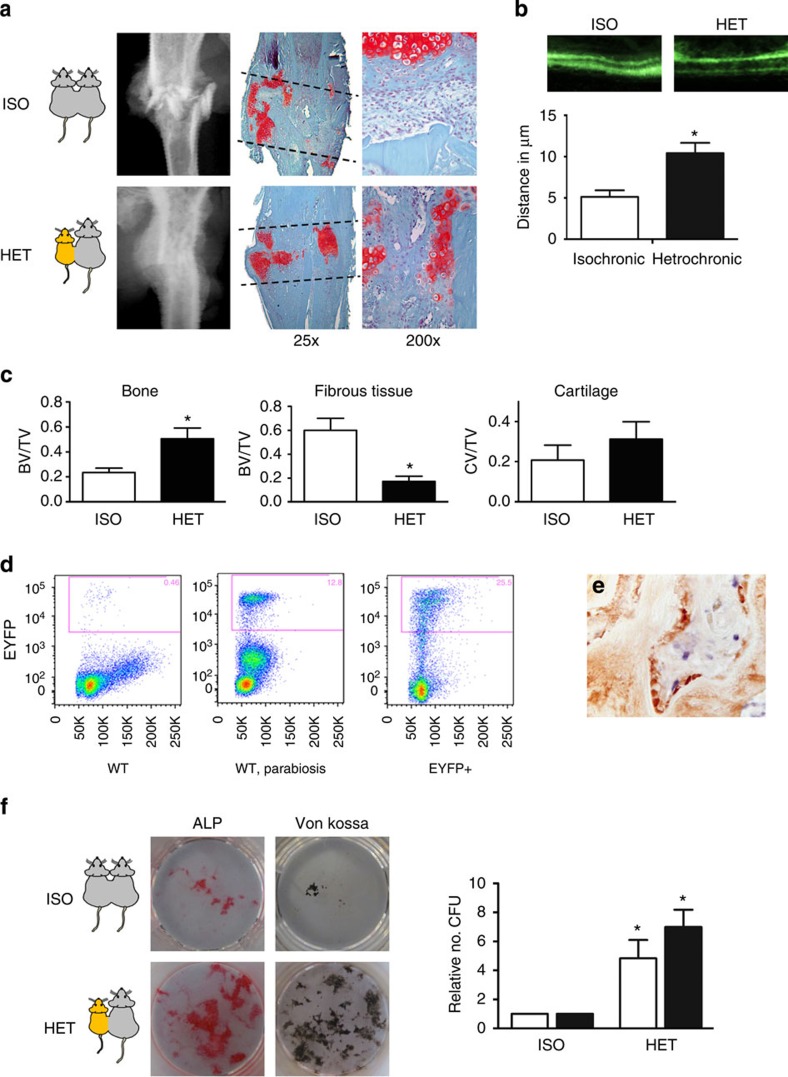
Exposure to a youthful circulation rejuvenates fracture repair and osteogenic potential in older animals. (**a**) Tibae of 20-month-old mice in isochronic (ISO) or heterochronic (HET) parabiotic pairs were fractured and harvested 14 days post injury. Radiographic and histologic (Safranin-O/Fast Green) analyses were used to investigate the progression of tissue repair (*n*=8 pairs ISO, *n*=11 pairs HET). Scale bars of × 25 images, 400 μm and of × 200 images, 100 μm. The fracture site is outlined by dashed lines. (**b**) Mineral apposition during fracture repair was analysed using calcein labelling (top). The distance between the dye fronts was quantified (bottom). (**c**) Amounts of bone, fibrous tissue and cartilage deposited in the fracture callus was quantified using histomorphometric analysis (five sections were analysed per fracture callus, *n*=8 fracture calluses-Iso, *n*=11 fracture calluses-Het). (**d**) Blood-sharing of parabiosis pairs was confirmed using FLOW cytometry by observing EYFP+ cells in the circulation of WT mice (*n*=4–7 mice per group). (**e**) Immunohistochemistry was used to identify EYFP+ cells (blue) and osteocalcin-expressing cells (brown) in the fracture callus. Scale bar, 50 μm. (**f**) Bone marrow stromal cells were aspirated from the tibae of unfractured 20-month-old mice in isochronic or heterochronic parabiotic pairs, adhered to tissue culture plastic and differentiated under osteogenic conditions. After 15 days in differentiation media, cultures were washed, fixed and stained for ALP or mineral (Von Kossa; *n*=5 triplicates per group). Differentiation potential of cultures was quantified by analysing the number of CFU for ALP (white bars) and Von Kossa (black bars; *n*=5 triplicates per group). Data are expressed as mean±95% confidence interval. *P<0.05, statistically significant (Dunnett's test).

The capacity for bone-fracture repair and osteoblastic differentiation potential diminish with age (Supplementary Fig. 1). Heterochronic parabiosis was used to study the effect of a youthful circulation on the pace and quality of tibial fracture repair in old animals. Mice expressed either enhanced yellow fluorescent protein (EYFP) ubiquitously or thymidine kinase (dTK) driven by a fragment of the type I collagen promoter uniquely expressed in osteoblasts. Expression of EYFP allowed for tracking of cells, while expression of dTK allowed for osteoblast ablation on treatment with ganciclovir^21^. Upon treatment with gancivlovir, osteoblasts are ablated from dTK+ mice and fracture repair is halted (Supplementary Fig. 2). Effective sharing of circulation in parabiotic pairs was demonstrated by detecting EYFP+ cells in the circulation of both mice (Fig. 1d). Isochronic (young–young or old–old) pairings and heterochronic (young–old) pairings of parabiosis were established. Heterochronic parabiosis led to rejuvenation of both fracture-repair phenotype and osteogenic potential in 20-month-old mice (Fig. 1a). Fracture calluses from old mice in heterochronic pairings contained twice the bone tissue and substantially less fibrotic tissue (fibrotic tissue indicated by arrows—Fig. 1a) than fracture calluses from old mice in isochronic parabiotic pairings (Fig. 1c). Interestingly, the amount of cartilaginous tissue deposited at the fracture site was relatively unchanged. Calcein labelling confirmed an increase in bone apposition in rejuvenated calluses (Fig. 1b). Immunohistochemical analysis of the fracture calluses was used to identify engrafted EYFP+ cells. EYFP+ cells (blue) in fracture calluses located to the marrow space at the site of injury (arrows); however, they were not associated with cells that formed new bone, nor did they differentiate to osteocalcin+ (brown) cells (Fig. 1e). BMSCs were differentiated to osteoblasts to investigate osteogenic potential. Bone marrow stromal cultures from old mice in heterochronic pairs contained more osteoblastic colonies (alkaline phosphatase; ALP) and produced higher levels of mineralization (Von Kossa; VK) than their isochronic counterparts (Fig. 1f).

Rescue of the aged phenotype was accomplished even when osteoblasts were ablated from the young parabionts in heterochronic pairings (Supplementary Fig. 3a). Conversely, when old mice in heterochronic pairs were depleted of osteoblasts, they were unable to undergo fracture repair or osteoblastic differentiation (Supplementary Fig. 3b). This suggests that endogenous osteoblasts are indispensible for fracture repair, and that circulating cells able to differentiate into osteoblasts are not responsible for rejuvenation. While others speculate that there may be a circulating^22^,^23^,^24^ or migrating^25^,^26^ osteogenic precursor cell, our data from parabiosis support the notion that such a migrant cell type does not play a major role in fracture repair^27^,^28^,^29^. Young mice in heterochronic parabiosis that were fractured showed a slight decrease in the amount of bone present at the fracture site, and *in vitro* osteogenic potential, but these changes were not significant (Supplementary Fig. 4). Together, these data indicate the existence of a circulating youth factor able to rejuvenate fracture repair and osteogenic potential in older mice. This rejuvenation is not dependent on osteoblasts residing in the parabiont partner, but rather arises from a circulating cell or molecule able to influence the endogenous, aged osteoblasts during this repair process.

### Young bone marrow rejuvenates aged bone healing

**Figure 2:**
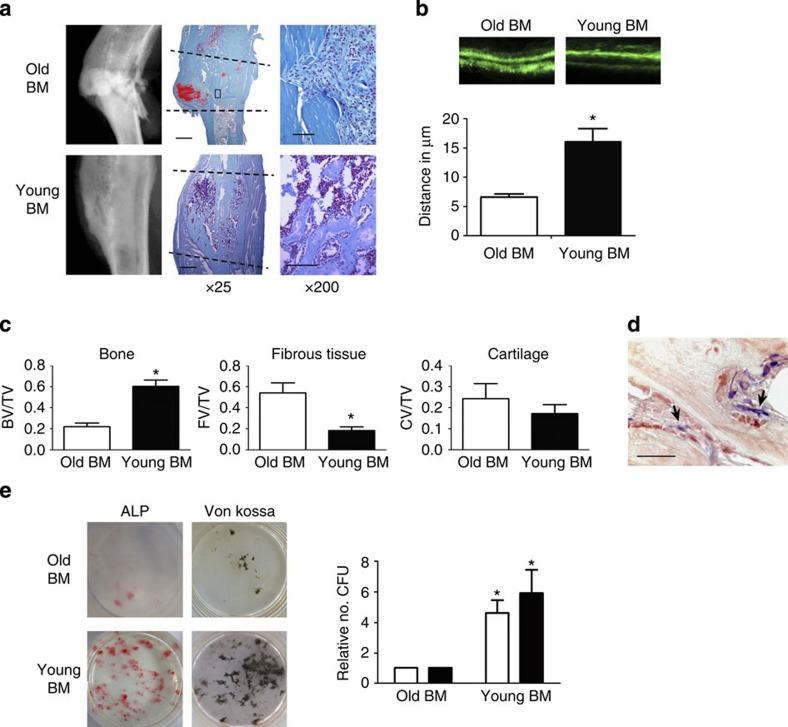
Engraftment of young bone marrow rejuvenates fracture repair and osteogenic potential in older animals. (**a**) Tibae of 20-month-old mice engrafted with old and young bone marrow were fractured and harvested 21 days post injury. Radiographic and histologic (Safranin-O/Fast Green) analyses were used to investigate the progression of tissue repair (*n*=10; Old BM, *n*=9; young BM). Scale bars of × 25 images, 400 μm and of × 200 images, 100 μm. The fracture site is outlined by dashed lines. (**b**) Mineral apposition during fracture repair was analysed using calcein labelling (top). The distance between the dye fronts was quantified (bottom). (**c**) Amounts of bone, fibrous tissue and cartilage deposited in the fracture callus was quantified using histomorphometric analysis (five sections were analysed per fracture callus, *n*=10 fracture calluses—Old BM, *n*=9 fracture calluses—Young BM). (**d**) Immunohistochemistry was used to identify EYFP+ cells (blue) and osteocalcin-expressing cells (brown) in the fracture callus. Scale bar, 50 μm. (**e**) Bone marrow stromal cells were aspirated from the tibae of unfractured 20-month-old mice engrafted with old and young bone marrow. Cells were adhered to tissue culture plastic and differentiated in osteogenic media. After 15 days in differentiation media, cultures were washed, fixed and stained for ALP or mineral (Von Kossa; *n*=5 triplicates per group). (**e**) Differentiation potential of cultures was quantified by analysing the number of CFU for ALP (white bars) and Von Kossa (black bars; *n*=5 triplicates per group). Data are expressed as mean±95% confidence interval. *P<0.05, statistically significant (Dunnett's test).

There are multiple potential sources for such a circulating factor, including hormones, small molecules, minerals or factors produced by haematopoietic lineage cells. To determine whether the rejuvenating factor originates from a haematopoietic cell, fracture repair and osteogenic potential were examined in mice after undergoing bone marrow transplantation. Engraftment of young bone marrow rescued fracture repair and osteogenic potential in aged animals to a similar degree as seen in heterochronic parabiosis (Fig. 2 and Supplementary Fig. 5). Rejuvenated fracture calluses contained more bone tissue and less fibrotic tissue (fibrotic tissue indicated by arrows—Fig. 2a) 21 days post fracture (Fig. 2c) and 14 days post fracture (Supplementary Fig. 5). Interestingly, neither the amount of cartilaginous tissue deposited at the fracture site nor osteoclast activity were altered by rejuvenation. Calcein labelling confirmed an increase in bone apposition in rejuvenated calluses (Fig. 2b). Together, these data confirm that rejuvenation of fracture repair through parabiosis or through bone marrow transplant is rooted in enhanced osteoblastic differentiation and bone formation. Conversely, we also found that old bone marrow decreased the fracture-repair capacity of young mice, albeit by an insignificant margin (Supplementary Fig. 6a). Immunohistochemical analysis of the fracture calluses was used to identify engrafted EYFP+ cells. Engrafted EYFP+ cells in the fracture calluses located to the injury site in the bone marrow (arrows); however, the EYFP+ cells (blue) did not differentiate to osteocalcin+, osteoblastic cells (brown; Fig. 2d). Thus, the mesenchymal cells directly contributing to the bone repair were not EYFP+, but rather the EYFP+ cells appear to locate in the vicinity of the bone-forming osteoblastic cells.

Similar to the findings in parabiosis, ablation of osteoblasts in host, dTK+ animals resulted in complete ablation of fracture repair and osteogenic potential, while ablation of donor osteoblasts had no effect on improved fracture repair phenotype (Supplementary Fig. 7). Thus, host osteoblastic cells are indispensible for bone formation, while donor cells contain little to no osteoblastic component. Interestingly, we observed that 100% of the cells engrafted into the bone marrow space were CD45+ haematopoietic cells, thus few if any mesenchymal progenitors are engrafted into the host animal (Supplementary Fig. 8). Radiographic and histologic analyses of the fracture calluses from old mice engrafted with young bone marrow indicated increased bone deposition and decreased fibrosis (Fig. 2a,c). Bone marrow stromal cell cultures of old mice engrafted with young bone marrow contained more robust ALP and VK staining than cultures of mice, which were engrafted with old bone marrow (Fig. 2e).

Rejuvenation of fracture repair through parabiosis and bone marrow transplantation led to a decreased amount of fibrosis and increased amount of bone tissue deposition while deposition of cartilage tissue remained unchanged, thus showing that the rejuvenation effects are due to enhanced osteoblastic differentiation. Multiple groups have demonstrated the reversal of bone age via bone marrow transplant^30^,^31^; here we establish that the rejuvenation is through non-osteoblastic CD45+ cells. These data recapitulate the findings of our parabiosis model and, taken together, show that haematopoietic cells, which have circulatory capacity but are not able to become osteoblasts, are responsible for the rejuvenation of aged fracture repair.

### Young bone marrow cells secrete a youth factor

**Figure 3:**
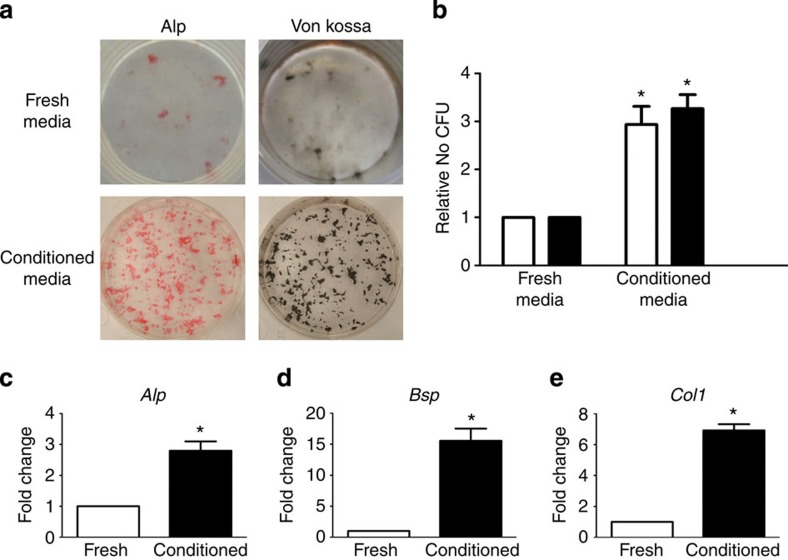
Media conditioned by young BMSCs rejuvenate aged osteogenic potential. (**a**) Bone marrow stromal cells were aspirated from the tibae of unfractured 20-month-old mice, adhered to tissue culture plastic and differentiated in osteogenic media. Cells were differentiated in fresh osteogenic media or in osteogenic media first conditioned by young bone marrow cells. After 15 days of differentiation, cultures were washed, fixed and stained for ALP or mineral (Von Kossa; *n*=9 triplicates per group). (**b**) Differentiation potential of cultures was quantified by analysing the number of CFU for ALP (white bars) and Von Kossa (black bars; *n*=5 triplicates per group). (**c**) After 10 days of differentiation, transcript levels of osteogenic markers were determined from old cells in fresh (white bars) and conditioned (black bars) osteogenic media (*n*=4 triplicates per group). Data are expressed as mean±95% confidence interval. *P<0.05, statistically significant (Dunnett's test).

To determine whether young bone marrow cells could alter the osteogenic potential of old BMSCs, conditioned media were examined. Media conditioned by young BMSCs were able to rescue the age-related decrease in osteogenic potential of old BMSCs. Rescued cells showed greater ALP and VK staining (Fig. 3a,b), and significantly higher levels of osteogenic markers (*Alp, Bsp, Col1*) relative to control (Fig. 3c). In contrast, media conditioned by old BMSCs did not alter the osteogenic potential of young BMSCs (Supplementary Fig. 9a). The transferred media did not contain cells or particulates but centrifugation-based isolation confirmed the rejuvenation factor to be greater than 10 kDa. Heat denaturation of the media abolished rejuvenation (Supplementary Fig. 9b) and media conditioned by young fibroblasts was not able to induce reversal of age. Our findings expand on previous data suggesting that young cells produce a matrix that enhances osteogenic differentiation^32^. We show that young bone marrow cells secrete a dissolved, transferable, heat-sensitive molecule that can rejuvenate osteoblast differentiation.

Bone marrow aspirates were used to generate BMSC cultures. These aspirates contain cells of mesenchymal and haematopoietic lineages; however, culture conditions used in this work guides differentiation for osteoblasts (mesenchymal differentiation) rather than haematopoietic differentiation. During this process, tissue culture plastic-adherent haematopoietic cells may remain attached to the culture surface. To determine the proportion of haematopoietic lineage cells in our BMSC ‘conditioning cultures', we used flow cytometry to identify the percentage of CD45+ cells. We found that as much as 50% of cells adhered to the tissue culture surface are CD45+ at the initiation of differentiation. Thus, in our *in vitro* studies, as well as in our animal work, young CD45+ cells are likely the source of the transferable factor that is able to rejuvenate aged osteoblastic differentiation.

### β-catenin levels are dysregulated with age

**Figure 4:**
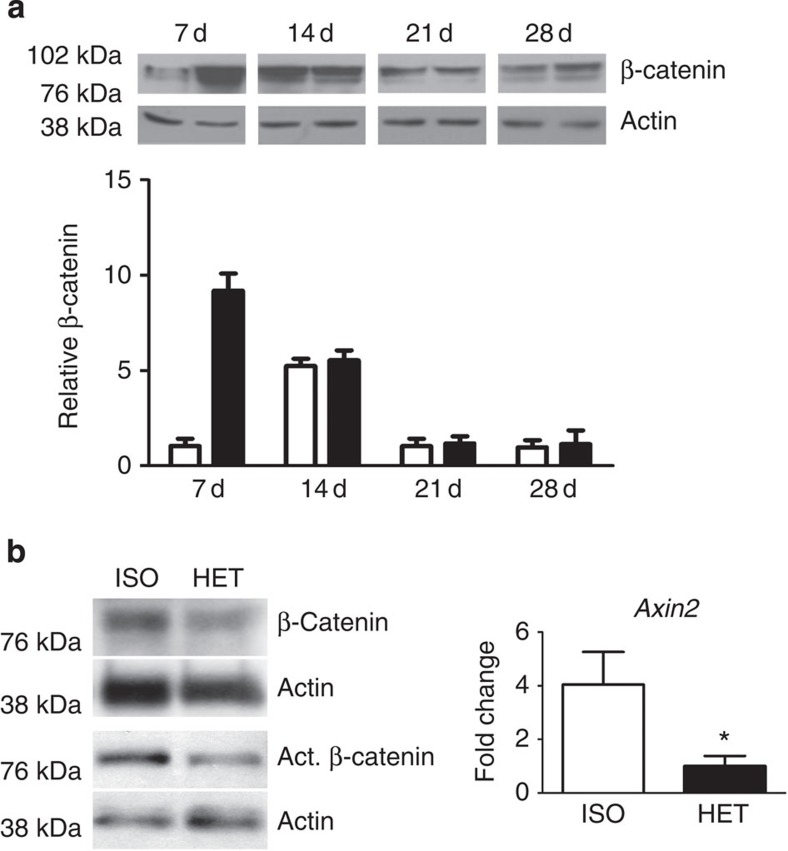
β-catenin levels are elevated in older mice but lowered through rejuvenation. (**a**) Fracture calluses from tibae of 4- and 20-month-old mice were investigated for β-catenin protein levels using western blot analysis. β-catenin levels were quantified relative to actin (loading control—white bars, young; black bars, old; *n*=5 fracture calluses per group). (**b**) Fracture calluses from 20-month-old mice in ISO or HET parabiotic pairs were investigated 7-days post fracture. Samples were investigated for β-catenin and active (unphosphorylated) β-catenin protein levels using western blot analysis (*n*=5 fracture calluses per group) and *Axin2* transcript levels using RT-PCR (*n*=4 fracture calluses per group). Data are expressed as mean±95% confidence interval. *P<0.05, statistically significant (Dunnett's test).

Temporal regulation of β-catenin is important for robust differentiation of mesenchyaml precursors to osteoblasts. Indeed, β-catenin levels are elevated during the early phases of bone fracture repair and dysregulation either to higher or lower levels inhibits normal healing. Dysregulation of β-catenin has been implicated in age-dependent changes in tissue regeneration^9^,^10^. Our analysis of unfractured tibae from old and young mice indicated there to be slightly higher levels of β-catenin in old tibae than in young tibae (Supplementary Fig. 10a). This modestly higher level of β-catenin at the onset of bone injury could translate into a propensity to differentiate towards a fibroblastic fate rather than an osteoblastic fate. TCF-Lef reporter mice showed higher levels of LacZ in old fracture calluses than in young calluses in the first week following a fracture (Supplementary Fig. 10b). Western blot analysis of young and old 7-day fracture calluses showed that β-catenin protein levels in old calluses were substantially higher than in those from young animals (Fig. 4a). β-catenin levels in fracture calluses from old mice reached a level nine times higher than that found in young mice, and this higher level is reached at an earlier time point (7 days in old mice, compared with 14 days in young mice; Fig. 4a).

These differences in the β-catenin level could have a critical effect on fracture repair. β-catenin levels are elevated at a time when cells are yet to commit to an osteochondral lineage, and an increase in β-catenin at this time point could lead to a shift from an osteoblastic to a fibroblastic phenotype. This is a potential cause for the high content of fibrotic tissue observed in the fracture calluses of old mice. Parabiosis-based rejuvenation lowered the β-catenin level of rejuvenated fracture calluses during early fracture repair (7 days post fracture) and is associated with decreased amounts of fibrotic tissue being deposited in the fracture callus. Mice in which the aged fracture repair was rejuvenated though heterochronic parabiosis showed a reduced level of total β-catenin, activated (unphosphorylated) β-catenin protein and *Axin2* transcript, a β-catenin target gene^33^, in the fracture calluses (Fig. 4b). This rejuvenation resulted in a tissue regeneration process that was less fibrotic and lead to greater bone deposition than in controls (Fig. 1). β-catenin levels of rejuvenated (*in vitro*), differentiating osteoblasts were lower (similar to young, differentiating BMSCs; Supplementary Fig. 10b). Together, these data show that β-catenin levels are elevated during early stages of fracture repair in aged mice, and that rejuvenation lowers the level towards that seen in younger animals.

### Modulation of β-catenin is required for rejuvenation

To confirm the importance of β-catenin modulation during rejuvenation, conditional β-catenin-stabilized and β-catenin-null mice were engrafted with young, EYFP+ bone marrow. The protein produced by the stabilized or null alleles of β-catenin lack the ability to be normally regulated by canonical Wnt signalling. In recipient mice expressing the stabilized or null β-catenin alleles, engraftment with young bone narrow cells did not alter the observed fracture repair phenotype, despite rejuvenation of the control animals (Supplementary Fig. 10d,e). These data confirm that modulation of β-catenin is required for rejuvenation of aged fracture repair and osteogenic potential.

### Decreased β-catenin early during injury rejuvenates healing

**Figure 5:**
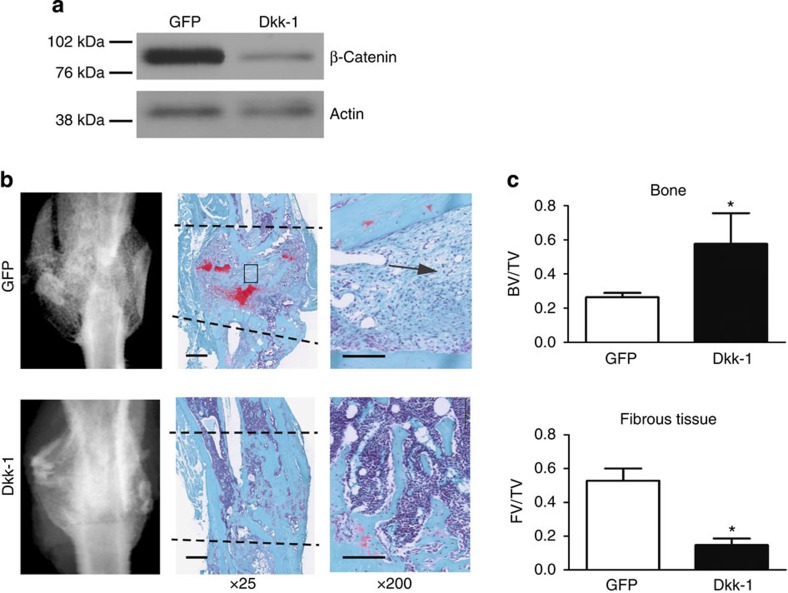
Modulation of β-catenin rejuvenates fracture repair. Tibae of 20-month-old mice were treated with adenovirus carrying GFP or Dkk-1, and fracture calluses were harvested 21 days post fracture. (**a**) Modulation of β-catenin 7 days post fracture was verified using western blot analysis. (**b**) Radiographic and histologic (Safranin-O/Fast Green) analyses were used to investigate the progression of tissue repair (*n*=7; GFP, *n*=11; Dkk-1). Scale bars of × 25 images, 400 μm and of × 200 images, 100 μm. The fracture site is outlined by dashed lines. (**c**) Amounts of bone and fibrotic tissue deposited in the fracture callus was quantified using histomorphometric analysis (five sections were analysed per fracture callus, *n*=7 fracture calluses-GFP, *n*=11 fracture calluses-Dkk-1). Data are expressed as mean±95% confidence interval. *P<0.05, statistically significant (Dunnett's test).

Age-dependent differences in β-catenin levels between old and young fracture calluses were greatest during early fracture repair (Fig. 4a). β-catenin levels during fracture repair are modulated by Wnt signalling and can be modulated by blocking Wnt receptor activity, for instance, by overexpression of Dkk-1 (refs 13, 14, 15). Dkk-1 is an extracellular protein that, when secreted by cells, can act on the local tissue environment as well as systemically. As such, treatment with Dkk-1 (refs 35, 36, 37) at the time of injury was used to assess the effect of decreasing β-catenin during early fracture repair. Western blot analysis confirmed a decrease in β-catenin on treatment with Ad-Dkk-1, relative to Ad-GFP (green fluorescent protein) control (Fig. 5a). Dkk-1 expression by the adenovirus was restricted to early stages of fracture repair. Western blot analysis confirmed expression 3 days post fracture and a loss of expression by 14 days post fracture (Supplementary Fig. 11). Radiographs and histological analysis (Safranin-O/Fast Green) of calluses 21-day post fracture confirmed improved healing with Dkk-1 treatment during the early stages of fracture repair (Fig. 5b). Dkk-1-treated calluses contained more bone content and less fibrotic tissue (indicated by arrows) than controls (Fig. 5c). While increased Wnt/β-catenin signalling has been shown to enhance bone density^20^, here we show the importance of temporal modulation of the Wnt/β-catenin pathway during bone tissue regeneration. Elevated levels of β-catenin during early fracture repair results in deposition of fibrosis tissue, while decreased levels of β-catenin in early fracture repair likely allowed progenitor cells to commit to an osteochondral lineage^10^,^14^. These data confirm that aged fracture repair can be rejuvenated through temporal modulation of the Wnt/β-catenin pathway. They also raise the possibility that pharmacologic agents for treating osteoporosis, which activate β-catenin might hinder healing in older patients.

## Conclusions

Here we demonstrated that aged fracture repair was rejuvenated through the modulation of β-catenin by exposure to a youthful circulation. Heterochronic parabiosis or engraftment of young bone marrow was able to rejuvenate bone repair and osteoblast differentiation. Within the population of bone marrow cells, CD45+ haematopoietic cells play an important role in this reversal of age. This rejuvenation was driven by a factor that was able to modulate Wnt/β-catenin signalling. Having demonstrated the necessity of β-catenin modulation, we also show that decreasing levels of β-catenin in old fracture calluses early in fracture repair can rejuvenate the fracture repair process.

Interestingly, these findings indicate a potential role for inflammation in rejuvenation of fracture repair. With age, this inflammatory response due to injury changes leads to increased fibrosis and inefficient tissue regeneration. In bone marrow-derived rejuvenation, only CD45+ haematopoietic cells were engrafted into the marrow space. These cells are responsible for the inflammatory response during injury and, as such, these cells likely result in a rejuvenated inflammatory response. Several studies, including work from our group, have confirmed the importance of an appropriate inflammatory response for effective fracture repair^37^,^38^,^39^. This raises the possibility that pharmacologic agents able to decrease Wnt/β-catenin signalling and/or modulate the inflammatory process could be administered in a temporal manner to improve the quality of fracture repair and even osseous integration of implants.

## Methods

### Transgenic mice

All mice were housed at the Toronto Centre for Phenogenomics and all animal use protocols were approved by the Animal Care Committee at the Toronto Centre for Phenogenomics. EYFP+ mice [129-Tg(Actb-EYFP)2Nagy] express enhanced yellow fluorescent protein under control of the ubiquitous chicken β-actin promoter^40^, and dTK+ mice express thymidine kinase under control of the osteoblast-specific, 2.3-kb, Col1a1 promoter^21^. EYFP and dTK mice were backcrossed together for eight generations before use. TCF-Lef reporter mice contain a LacZ gene located downstream of a c-fos promoter and three consensus TCF-binding sites. Functional signalling of β-catenin led to expression of β-galatosidase protein. β-catenin stabilized mice contain loxP sequences flanking exon 3. Cre-recombinase-based excision results in a stabilized form of β-catenin protein^41^. β-catenin null mice contain loxP sites in intron 1 and intron 6. Cre-recombinase-based excision results in nullification of this gene^42^. β-catenin stabilized and β-catenin null mice were crossed with Tg(CAC-cre/Esr1*)5AMC/J mice, which express Cre-recombinase in a ubiquitous chicken β-actin promoter. Cre-recombinase is restricted to the cytoplasm; however, treatment with tamoxifen allows localization to the nucleus, leading to recombination of the target gene. Tamoxifen was administered using two intraperitoneal injections per week (100 mg kg^−1^) beginning 1 week before and for the duration after fracture. Recombination of the conditional alleles was observed to be ∼80% at the fracture sites, confirming our previous observations^43^. ‘Old' mice are 20 months of age at the time of fracture or cell harvest, while ‘Young' mice are 4 months old.

### *In vivo* temporal modulation of β-catenin

For temporal decrease in β-catenin, 2 × 10^8^ p.f.u. of adenovirus harbouring Dickkopf-1 cDNA tagged with a C-terminal hexa-His tag or adenovirus harbouring GFP was injected at the fracture site 1-day pre-fracture^15^.

### Calcein labelling

Fluorochrome labelling was used to investigate active bone deposition. Calcein green (30 mg kg^−g^) in saline was delivered using peritoneal injections at 7 and 2 days before harvest of the fracture callus. Specimens were fixed in 70% ethanol and embedded in methylmethacrylate. Sections were then cut to 5 μm, and fluorescent microscopy imaging (495 nm/521 nm, fluorescein isothiocyanate) was used to observe the dye and the distance between the dye fronts was measured.

### Parabiosis surgery

Anastomosis surgery was carried out using 3- or 19-month-old female mice, 4 weeks before tibial fracture. Isochronic (young–young or old–old) and heterochronic (young–old or old–young) pairs were established. A shared blood supply was ensured 3 weeks after surgery using FLOW cytometry^44^.

### Bone marrow transplantation

Two- or sixteen-month-old mice were irradiated (900cG) and tail vein-injected with bone marrow isolated from 2- or 16-month-old mice (1 × 10^6^ cells in 200 μl PBS). Engraftment into the bone marrow was allowed to occur for 2 months and verified using FLOW cytometry.

### Tibial fracture model

Fractures were induced as previously described^43^. Briefly, mice tibiae were stabilized by inserting a 0.7-mm insect pin into the medullary cavity and were then fractured mid-shaft. After harvest, fracture calluses were either fixed in 10% buffered formalin for histology or microdissected from the surrounding normal bone and placed directly into liquid nitrogen for subsequent RNA or protein extraction.

### Fracture-callus analysis

Fractured tibiae were placed into formalin for 7 days and analysed using X-ray (Faxitron MX20). EDTA (20%; pH 8.0) was used to decalcify bone for 4 weeks with two solution changes per week. Calluses were then embedded in paraffin and sectioned at 5 μm in thickness. Samples were stained using Safranin-O and Fast Green. Here red staining confirms the presence of proteoglycans that indicates the deposition of cartilagenous tissue. Representative sections from a minimum of five fractured limbs were analysed using computer-assisted histomorphometry analysis and results were presented as percent of bone, fibrous or cartilagenous tissue deposited relative to fracture callus.

### BMSC culture

BMSCs were aspirated from tibiae of unfractured mice, and progenitor cells were isolated on the basis of their adherence to tissue culture plastic. Cells were plated in plating media (AMEM, 10% FBS, 100 U ml^−l^ Penn/Strep) for 7 days. Colony-forming units (CFUs) were defined as individual clusters of stained cells or matrix. To determine CFU-F, wells were washed with PBS, fixed with 10% formalin and stained with 0.25% crystal violet. For osteogenic differentiation, the media was changed to osteogenic media (plating media with 30 μM ascorbic acid, 8 mM phosphate and 10 nM dexamethasone). The first day in osteogenic media was considered Day 1, and media were changed every 2 days thereafter. In conditioned media experiments, media was transferred directly from well to well after two days of conditioning. For heat denaturation, media was heated to 95 °C for 10 min and then cooled to 37 °C. After 15 days in differentiation media, cells were stained with ALP (indicate osteoblasts), Alizaren Red (indicate mineral formation) or VK (indicate mineral formation).

### Real-time PCR and western blot analyses

Total RNA or protein was extracted from fracture calluses after dissection. The proximal and distal borders of the fracture callus are roughly outlined by dashed lines in the low-magnification histological images. For real-time PCR, cDNA template was generated using random hexamers and data were related to the transcript of ribosomal protein 18S as a housekeeping control. Primers for *Alp* (Mm00475834_m1), *BSP* (Mm00492555_m1) and *Col1* (Mm00801666_g1) were purchased from Applied Biosystems. For western blot analysis, indicated antibodies were detected using horseradish peroxidase-conjugated secondary antibodies and enhanced chemiluminescence (ECL). Relative band intensities were quantified using the ImageJ software ( http://imagej.nih.gov/ij/). Anti-β-catenin antibody (working concentration of 1.0 μg ml^−l^—06-734) was purchased from Millipore, anti-active β-catenin antibody (working concentration of 1.5 μg ml^−1^—05-665) was purchased from Millipore, anti-β-actin antibody (working concentration of 0.5 ng ml^−1^—CP01) was purchased from Calbiochem and anti-His antibody (working concentration of 0.2 ng ml^−1^—s-804) was purchased from Santa Cruz. Full western blot images are shown in Supplementary Fig. 12.

### Statistical analysis

Data are expressed as mean±95% confidence interval. One-way analysis of variance was performed followed by Dunnett's test when significant differences were detected. The data were considered to be statistically significant at a confidence level of 95% (*P*<0.05).

## Additional information

**How to cite this article:** Baht, G. S. *et al*. Exposure to a youthful circulaton rejuvenates bone repair through modulation of β-catenin. *Nat. Commun.* 6:7131 doi: 10.1038/ncomms8131 (2015).

## Supplementary Material

Supplementary Information Supplementary Figures 1-12
