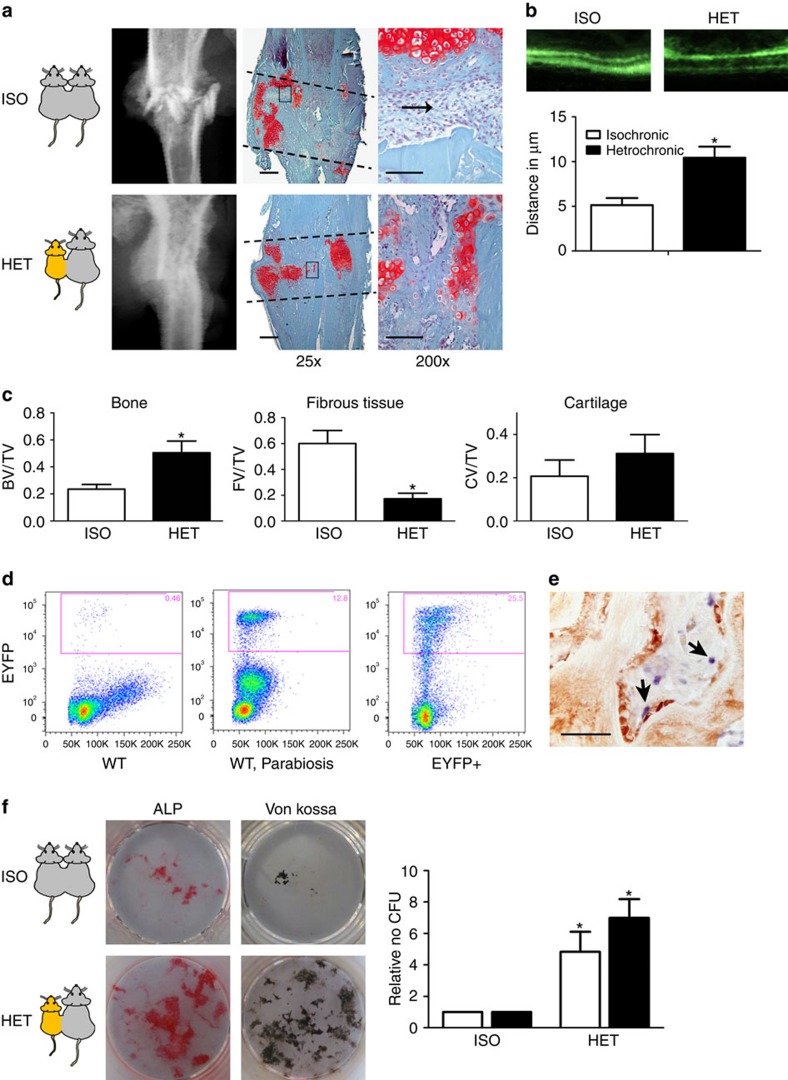# Erratum: Exposure to a youthful circulation rejuvenates bone repair through modulation of β-catenin

**DOI:** 10.1038/ncomms8761

**Published:** 2015-08-26

**Authors:** Gurpreet S. Baht, David Silkstone, Linda Vi, Puviindran Nadesan, Yasha Amani, Heather Whetstone, Qingxia Wei, Benjamin A. Alman

Nature Communications 6, Article number: 7131 10.1038/ncomms8131 (2015); Published 05192015; Updated: 08262015.

The original version of this Article contained an error in the spelling of circulation in the title of the paper, and in Fig. 1e, a scale bar and arrows were inadvertently omitted. These errors have now been corrected in both the PDF and HTML versions of the article.

## Figures and Tables

**Figure 1:**