# Supplementary material for: Exposure to a youthful circulaton rejuvenates bone repair through modulation of β-catenin
Source: Nat Commun. 2015 May 19;6:7131. doi: 10.1038/ncomms8131 (PMC4479006; doi:10.1038/ncomms8131)
Supplement: Supplementary Information — Supplementary Figures 1-12 [file ncomms8131-s1.pdf]

Supplementary Figure 1

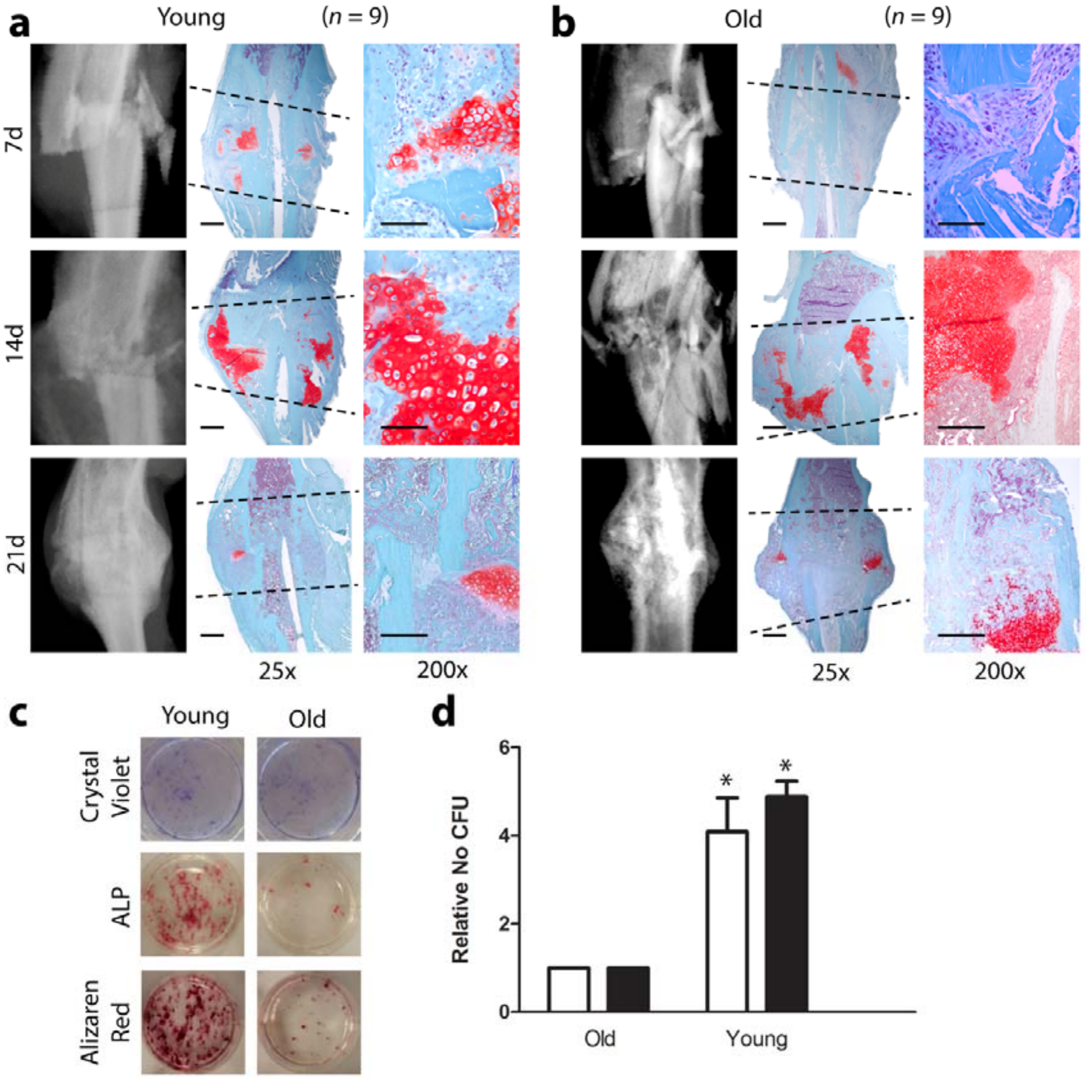

Supplementary Figure 1 – Aged mice display a diminished fracture-repair capacity and osteogenic potential. Tibae of **a,** 4- and **b,** 20-month old mice were fractured and harvested 7-, 14-, and 21-days post injury. Radiographic and histologic (Safranin-O/Fast Green) analysis were used to investigate the progression of tissue repair. Scale bars of 25x images represent 400µm and of 200x images represent 100µm. The fracture site is outlined by dashed lines. **c,** Bone marrow stromal cells were aspirated from the tibae of unfractured mice, adhered to tissue culture plastic, and differentiated under osteogenic conditions. After 7 days in plating media, cells were either stained with crystal violet to determine CFU-F (no significant difference) or grown in differentiation media for 15 days and stained for alkaline phosphatase (ALP) and mineral (Von Kossa). **d,** CFU-ALP (white bars) and CFU-VK (black bars) counts were determined from cultures of young and old BMSC's. Data are expressed as mean +/- 95% confidence interval. *Statistically significant, $p < 0.05$ (Dunnett's test).

Supplementary Figure 2

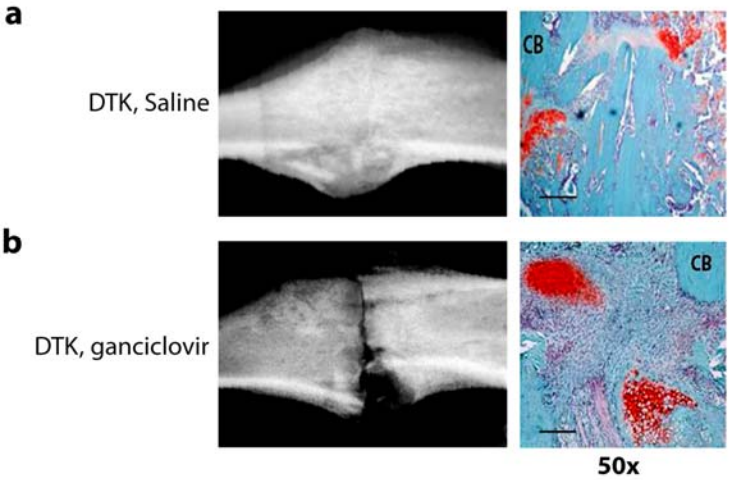

Supplementary Figure 2 – Ablation of osteoblasts results in an absence of fracture repair. DTK+/- mice were fractured and treated with **a,** saline or **b,** ganciclovir for the duration of the repair process. Fracture calluses were harvested 21-days post fracture. Radiographic and histologic (Safranin-O/Fast Green) analysis were used to investigate the progression of tissue repair. Scale bars represent 400µm.

Supplementary Figure 3

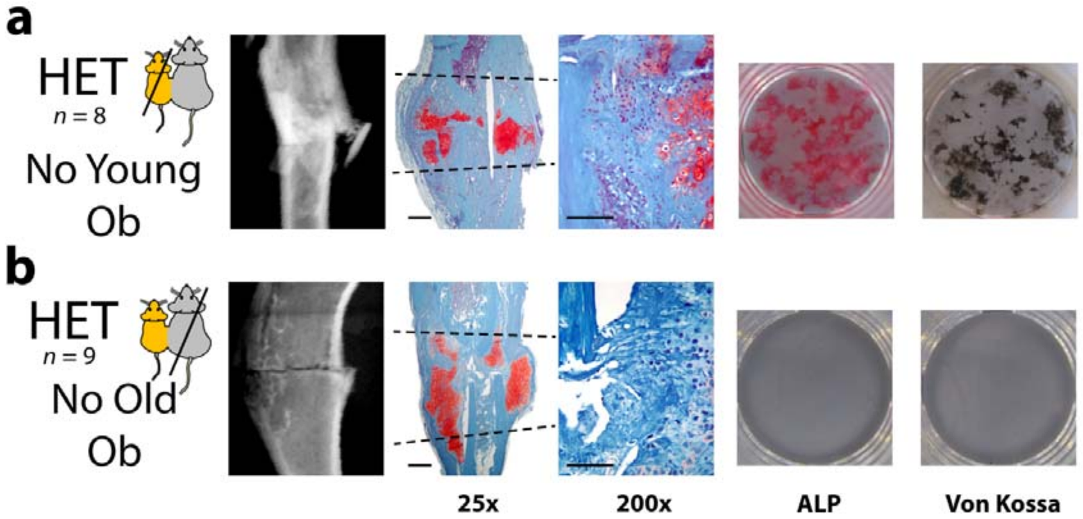

Supplementary Figure 3 – Ablation of young partner osteoblasts has no effect on rejuvenation but endogenous osteoblasts are required for parabiosis-based rejuvenation. Tibae of 20-month old mice in heterochronic parabiotic pairs were fractured and harvested 14-days post injury. During fracture healing the osteoblasts from either the **a,** young partner animal or **b,** the old fractured animal were ablated. Radiographic and histologic (Safranin-O/Fast Green) analysis were used to investigate the progression of tissue repair. Scale bars of 25x images represent 400µm and of 200x images represent 100µm. The fracture site is outlined by dashed lines. Bone marrow stromal cells were aspirated from the tibae of unfractured 20-month old mice in isochronic or heterochronic parabiotic pairs, adhered to tissue culture plastic, and differentiated under osteogenic conditions. After 15 days in differentiation media, cultures were washed, fixed, and stained for alkaline phosphatase (ALP) or mineral (Von Kossa).

Supplementary Figure 4

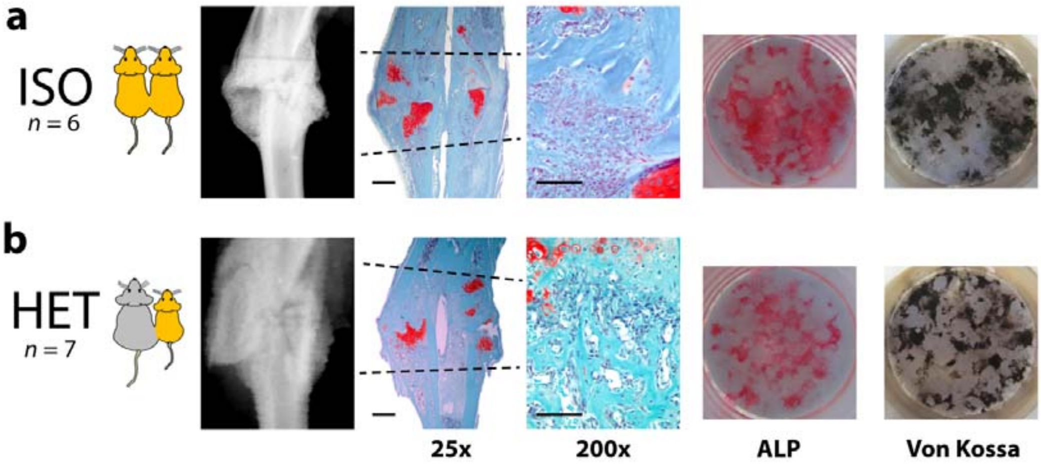

Supplementary Figure 4 – Young animals retain their robust capacity for fracture repair and osteoblast differentiation. Tibae of 4-month old mice in **a,** isochronic or **b,** heterochronic parabiotic pairs were fractured and harvested 14-days post injury. Radiographic and histologic (Safranin-O/Fast Green) analysis were used to investigate the progression of tissue repair. Scale bars of 25x images represent 400μm and of 200x images represent 100μm. The fracture site is outlined by dashed lines. Bone marrow stromal cells were aspirated from the tibae of unfractured 4-month old mice in isochronic or heterochronic parabiotic pairs, adhered to tissue culture plastic, and differentiated under osteogenic conditions. After 15 days in differentiation media, cultures were washed, fixed, and stained for alkaline phosphatase (ALP) or mineral (Von Kossa).

Supplementary Figure 5

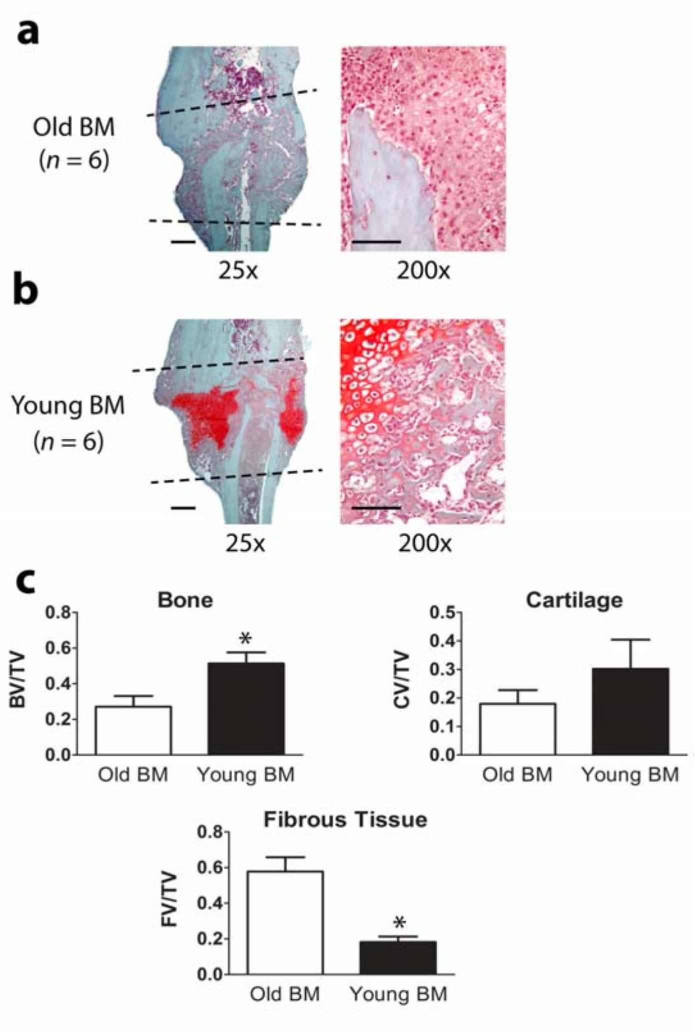

Supplementary Figure 5 – Engraftment of young bone marrow rejuvenates fracture repair and osteogenic potential in older animals. Tibae of 20-month old mice engrafted with **a,** old and **b,** young bone marrow were fractured and harvested 14-days post injury. Radiographic and histologic (Safranin-O/Fast Green) analysis were used to investigate the progression of tissue repair. Scale bars of 25x images represent 400µm and of 200x images represent 100µm. The fracture site is outlined by dashed lines. **c,** Amounts of bone, fibrous tissue, and cartilage deposited in the fracture callus was quantified using histomorphometric analysis (5 sections were analysed per fracture callus). Data are expressed as mean +/- 95% confidence interval. *Statistically significant, $p < 0.05$ (Dunnett's test).

Supplementary Figure 6

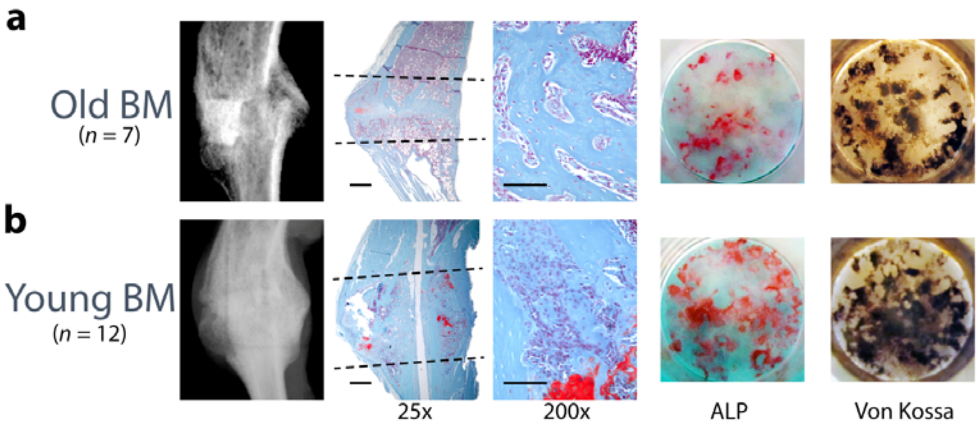

Supplementary Figure 6 – Young mice retain robust capacity for fracture repair and osteoblast differentiation. Tibae of 4-month old mice engrafted with **a,** old or **b,** young bone marrow were fractured and harvested 21-days post injury. Radiographic and histologic (Safranin-O/Fast Green) analysis were used to investigate the progression of tissue repair. Scale bars of 25x images represent 400µm and of 200x images represent 100µm. The fracture site is outlined by dashed lines. Bone marrow stromal cells were aspirated from the tibae of unfractured mice, adhered to tissue culture plastic, and differentiated under osteogenic conditions. After 15 days in differentiation media, cultures were washed, fixed, and stained for alkaline phosphatase (ALP) or mineral (Von Kossa).

Supplementary Figure 7

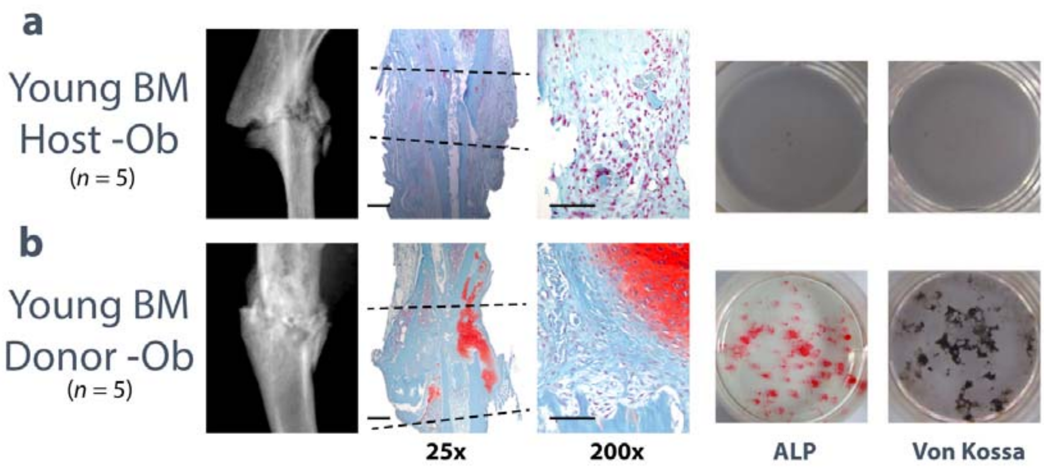

Supplementary Figure 7 – Ablation of young donor osteoblasts has no effect but endogenous osteoblasts are required for rejuvenation. Tibae of 20-month old mice engrafted with young bone marrow were fractured and harvested 21-days post injury. During fracture healing osteoblasts from either the **a,** host or **b,** the donor animal were ablated. Radiographic and histologic (Safranin-O/Fast Green) analysis were used to investigate the progression of tissue repair. Scale bars of 25x images represent 400µm and of 200x images represent 100µm. The fracture site is outlined by dashed lines. Bone marrow stromal cells were aspirated from the tibae of unfractured mice, adhered to tissue culture plastic, and differentiated under osteogenic conditions. After 15 days in differentiation media, cultures were washed, fixed, and stained for alkaline phosphatase (ALP) or mineral (Von Kossa).

Supplementary Figure 8

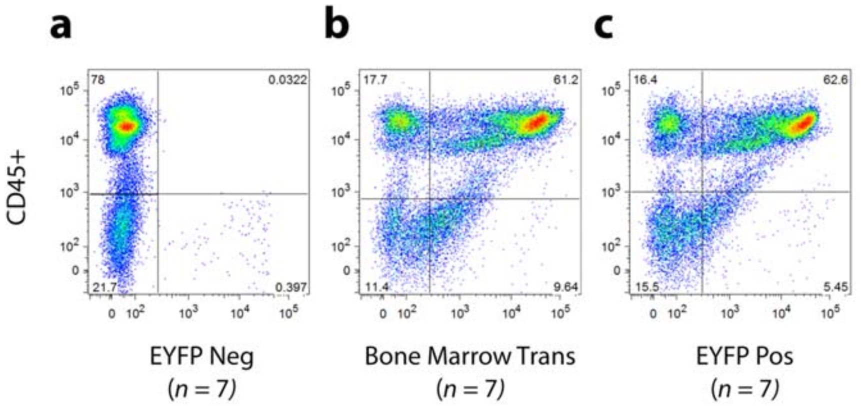

Supplementary Figure 8 – Engrafted bone marrow replaces the endogenous CD45+ cell population. Bone marrow from EYFP+/+ animals was engrafted into wildtype animals and investigated using flow cytometry for EYFP+, CD45+ cells.

Supplementary Figure 9

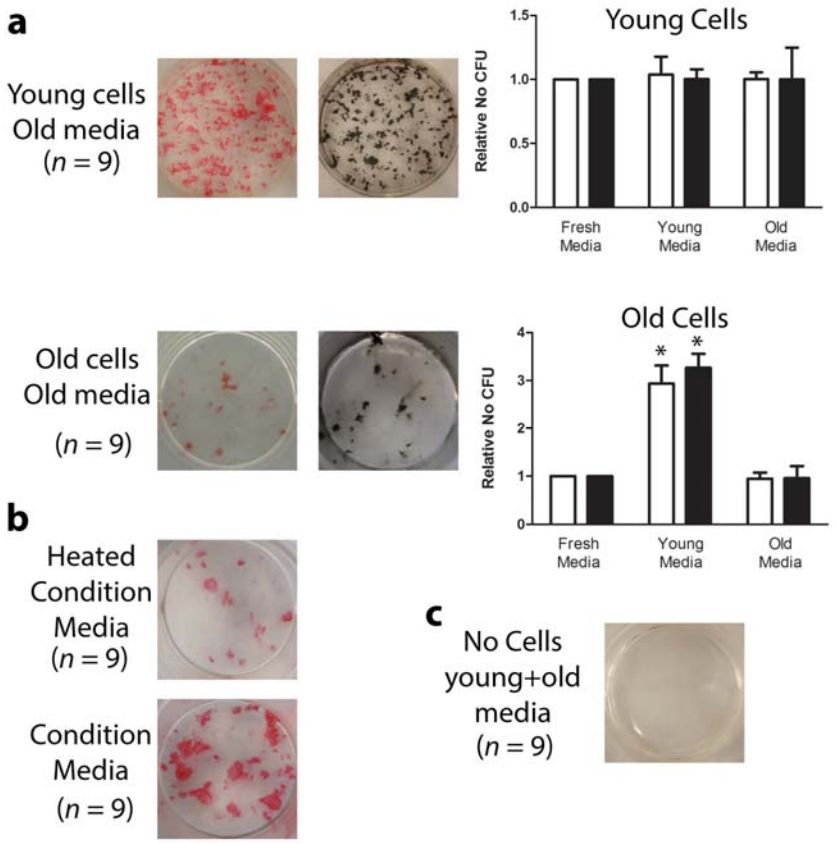

Supplementary Figure 9 – The rejuvenation factor contained within conditioned media is heat-sensitive. a, Bone marrow stromal cells were aspirated from the tibae of unfractured 4- and 20-month old mice, adhered to tissue culture plastic, and differentiated in osteogenic media conditioned by old cells. After 15 days of differentiation, cultures were washed, fixed, and stained for alkaline phosphatase (ALP) or mineral (Von Kossa). Differentiation potential of cultures was quantified by analysing the number of colony forming units (CFU) for ALP (white bars) and Von Kossa (black bars). **b,** Cells from 20-month old mice were aspirated and adhered to tissue culture plastic. Differentiation media was conditioned by young cells and either heated or untreated before use. After 15 days of differentiation, cultures were washed, fixed, and stained for alkaline phosphatase (ALP). **c,** Combined young- and old-conditioned media was incubated on tissue culture plastic and wells were stained for ALP. Data are expressed as mean +/- 95% confidence interval. * Statistically significant, $p < 0.05$ (Dunnett's test).

Supplementary Figure 10

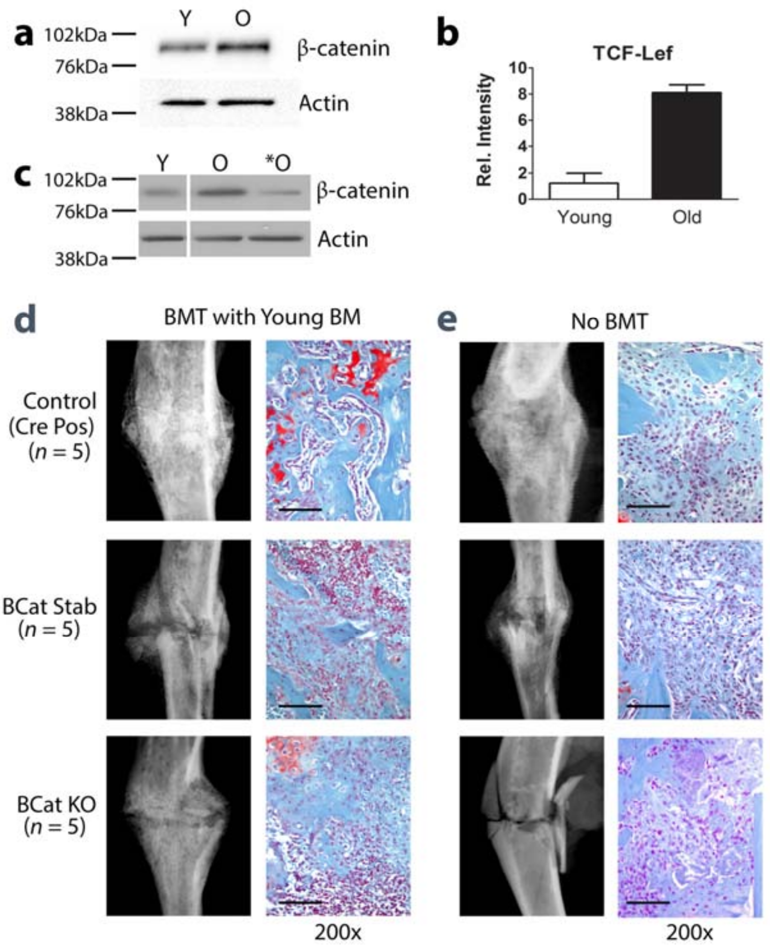

Supplementary Figure 10 – Modulation of β-catenin is required for rejuvenation of fracture repair. **a,** Unfractured tibiae from old mice and young mice were investigated for β-catenin levels. **b,** TCF-Lef reporter mice were fractured and fracture calluses were harvested 7-days post fracture. LacZ/β-galactosidase staining in the fracture callus was quantified. **c,** β-catenin levels were investigated in osteoblastic cultures of young cells (Y), old cells (O), and old cells rejuvenated with conditioned media (*O). **d,** 20-month old Cre-only control, β-catenin stabilized, and β-catenin null mice were engrafted with young bone marrow. After two months of engraftment, the tibiae of the mice were fractured and calluses were harvested at 21-days post fracture. Radiographic and histologic (Safranin-O/Fast Green) analysis were used to investigate the progression of tissue repair. Scale bars of images represent 100µm. Results were compared to **e,** 20-month old Cre-only control, β-catenin stabilized, and β-catenin null mice which did not undergo bone marrow transplantation.

Supplementary Figure 11

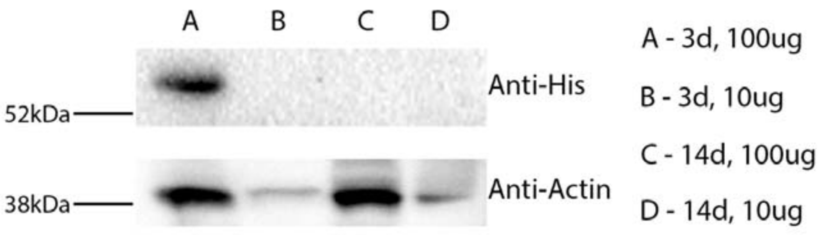

Supplementary Figure 11 – Ad-Dkk-1 protein is expressed during early stages of fracture repair. The tibiae of old mice were treated with Dkk-1-expressing adenovirus and fractured. Fracture calluses were harvested 3- and 14-days post injury. Expression of His-tagged Ad-Dkk-1 was confirmed by investigating protein lysates of fracture calluses using anti-His antibody.

Supplementary Figure 12

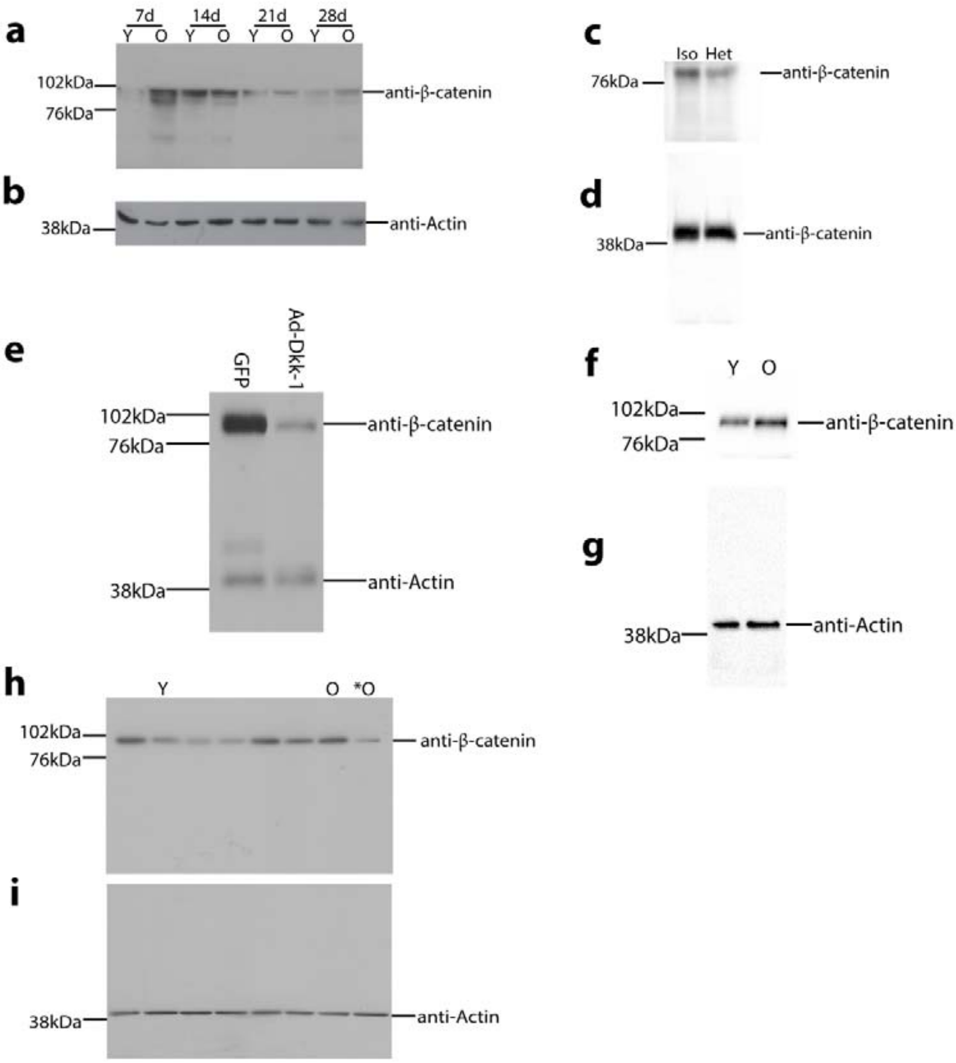

Supplementary Figure 12 – Full Western Blots. a, Tibial fracture calluses from young and old mice, anti-β-catenin. **b,** Tibial fracture calluses from young and old mice, anti-Actin. **c,** Tibial fracture calluses from parabiosis pairs, anti-β-catenin. **d,** Tibial fracture calluses from parabiosis pairs, anti-Actin. **e,** Tibia fracture calluses from Dkk-1-adenovirus-infected tibiae, anti-β-catenin & anti-Actin. **f,** Unfractured tibiae from young and old mice, anti-β-catenin. **g,** Unfractured tibiae from young and old mice, anti-Actin. **h,** Cell lysates from osteogenic BMSC culture, anti-β-catenin. **i,** Cell lysates from osteogenic BMSC culture, anti-Actin.
